# Descriptive summary of fatal work-related injuries, Western States, 2011–2017

**DOI:** 10.1080/15459624.2024.2302470

**Published:** 2024-02-26

**Authors:** Gialana Dang, Suzanne Marsh, Tristan Victoroff, Christa Hale, Joanna Watson, Kyle Moller, Laura Styles, Emily Healy, Tasha Chapman, Ketki Patel, Anna Fondario, Todd Schoonover, Sara Wuellner, Meredith Towle

**Affiliations:** a National Institute for Occupational Safety and Health (NIOSH), Denver, CO;; b NIOSH, Morgantown, WV;; c NIOSH, Spokane, WA;; d Centers for Disease Control and Prevention, Atlanta, Georgia;; e NIOSH, Atlanta, GA;; f California Department of Public Health, Sacramento, California;; g Montana Nonprofit Association, Helena, Montana;; h Oregon Department of Consumer and Business Services, Salem, Oregon;; i Texas Department of State Health Services, Austin, Texas;; j Utah Department of Health, Salt Lake City, Utah;; k Washington State Department of Labor and Industries, Olympia, Washington;; l Wyoming Workforce Services, Cheyenne, Wyoming

**Keywords:** Census of Fatal Occupational Injuries (CFOI), National Institute for Occupational Safety and Health (NIOSH), occupational fatalities, occupational safety

## Abstract

Work-related deaths are a persistent occupational health issue that can be prevented. However, prevention opportunities can be hampered by a lack of adequate public health resources. The Western States Occupational Network (WestON) is a network of federal, state, and local occupational health professionals that includes a 19-state region of the United States. To encourage public health collaboration, WestON partners examined work-related fatalities within the region. Fatality counts (numerators) were obtained from the U.S. Bureau of Labor Statistics (BLS) Census of Fatal Occupational Injuries restricted-access research files for all workers ages ≥15years and fatally injured in WestON states from 2011 through 2017. Estimates of full-time equivalent hours worked (FTE) (denominators) were retrieved from the BLS Current Population Survey. Annual average fatality rates were calculated as number of fatalities per 100,000 FTE over the study period. Rates were stratified by state, select demographics, industry sector, and event/exposure types. Pearson chi-squared tests and rate ratios with 95% confidence probability limits were used to assess rate differences. All analyses were conducted using SAS v.9.4. From 2011 through 2017, the annual average overall occupational fatality rate for the WestON region was 3.5 fatalities per 100,000 FTE, comparable to the overall U.S. fatality rate. Male workers had a fatality rate almost 10 times higher than female workers in the region. Fatality rates increased with successive age groups. Alaska and New Mexico had significantly higher fatality rates for all racial/ethnic groups compared to respective regional rates. Wyoming, North Dakota, and Montana had the three highest occupational fatality rates among foreign-born workers. Agriculture/forestry/fishing, mining/oil/gas extraction, and transportation/warehousing/utilities were industry sector groups with the three highest fatality rates regionally. Transportation-related incidents were the most frequent event type associated with occupational fatalities for all 19 states. Work-related fatalities are a crosscutting occupational public health priority. This analysis can be an impetus for collaborative multistate initiatives among a dynamic and varied occupational public health network to better meet the needs of a rapidly changing workforce.

## Introduction

During 2011–2017, an average of 4,843 workers died on the job each year in the United States, a rate of 3.3–3.6 fatalities per 100,000 full-time-equivalent hours worked (FTE) (BLS 2011–2017). Work-related deaths can be prevented; however, prevention activities can often be hampered by competing priorities, limited funding for state and local health departments, a shortage of occupational safety and health (OSH) professionals, and lack of training opportunities (CSTE 2018). Regional public health collaborations can maximize programmatic impact in the context of limited resources. For example, regional-level data analysis can highlight patterns of workplace fatalities across states, industries, and demographic groups and identify common challenges.

The Western States Occupational Network (WestON) is one of several regional occupational health networks in the United States (CSTE 2008). This network was established in 2008 to build state-level OSH capacity among 19 states (Alaska, Arizona, California, Colorado, Hawaii, Idaho, Kansas, Montana, Nebraska, Nevada, New Mexico, North Dakota, Oklahoma, Oregon, South Dakota, Texas, Utah, Washington, and Wyoming). Members include epidemiologists from the 19 WestON states, federal partners from the National Institute for Occupational Safety and Health (NIOSH), the Department of Labor (DOL) Occupational Safety and Health Administration (OSHA), and professionals from NIOSH Education and Research Centers and NIOSH Centers for Agricultural Safety and Health.

To encourage regional collaboration and identify common occupational health priorities, NIOSH and WestON state partners examined patterns in fatal work-related injuries in the WestON region over a 7-year period by select population demographics, employment characteristics, event/exposure types, and industry sectors.

## Methods

Data for this project were provided through a memorandum of understanding allowing NIOSH access to restricted-access research files from the U.S. Bureau of Labor Statistics (BLS) Census of Fatal Occupational Injuries (CFOI) for the years 2011–2017. In 2020, BLS changed its data reporting policies related to use of the restricted-access research files that prevented including data from after 2017 in this study. Fatality counts were obtained for all workers ages 15 years and older and fatally injured in WestON states from 2011 through 2017. For CFOI, BLS considers a fatal injury to be work related if the person was self-employed, working for pay, or volunteering; engaged in a legal work activity; and was present at or traveling between the work site(s) or traveling as part of job requirements at the time of the event. Fatalities that occur during a person’s normal commute to and from work are not included in CFOI. CFOI includes fatalities occurring in public- or private-sector employment situations regardless of establishment size (BLS 2012a).

For this analysis, workers ages 15 and older were selected because denominator data used for rate calculations were only available for workers ages 15 years and older. State of fatal injury was used to select WestON region cases from CFOI. Event or exposure leading to fatal injury in this analysis was defined using the BLS Occupational Injury and Illness Classification System (OIICS) v.2.01 (BLS 2012b). Industry classifications for data from 2011 through 2013 used the 2007 version of the North American Industrial Classification System (NAICS) codes (OMB 2007); data from 2014 through 2017 used the 2012 version (OMB 2012). Data were stratified at broad event/exposure categories and industry sector levels. Race and ethnicity were combined into five broad groups: non-Hispanic White, non-Hispanic Black, non-Hispanic American Indian/Alaska Native/Asian/Pacific Islander (AI/AN/AAPI), non-Hispanic other race (including more than one race), and Hispanic. Workers of Hispanic ethnicity might be of any race or combination of races. Foreign-born status was determined by place of birth and whether parents were U.S. citizens at time of birth. Workers born in the United States or its territories (including Puerto Rico, Guam, and the United States Virgin Islands) or abroad to a U.S. citizen parent or parents were defined as U.S. born. Workers who were foreign-born included anyone not a U.S. citizen at birth, including naturalized citizens and noncitizens.

Estimates of full-time-equivalent hours worked (FTE) were retrieved from the BLS Current Population Survey (CPS). Using fatality counts as numerators and FTE as denominators, annual average fatality rates were calculated as number of fatalities per 100,000 FTE hours worked over the 7-year study period and stratified by state, select demographics, industry sectors, and event and exposure types.

All occupational fatality rates reflect an annual average rate calculated over the 7-year study period. Pearson chi-squared tests were used to assess differences in annual average fatality rates over the 7-year period between individual states and the overall rates of the 19-state WestON region. Rate ratios (RR) with 95% confidence intervals (CI) were calculated to compare individual state rates with the corresponding WestON regional rate stratified by sex, age group, race/ethnicity, U.S. versus foreign-born status, industry sector, and event/exposure type. Rate ratios were also used to compare rates within states by race/ethnicity, with non-Hispanic White workers used as the referent group, and U.S.-born versus foreign-born worker status, with U.S.-born workers used as the referent group. All significance tests for rate comparisons used a 95% confidence probability limit (*p* < 0.05). All analyses were conducted using SAS version 9.4.

## Results

**Figure 1.**
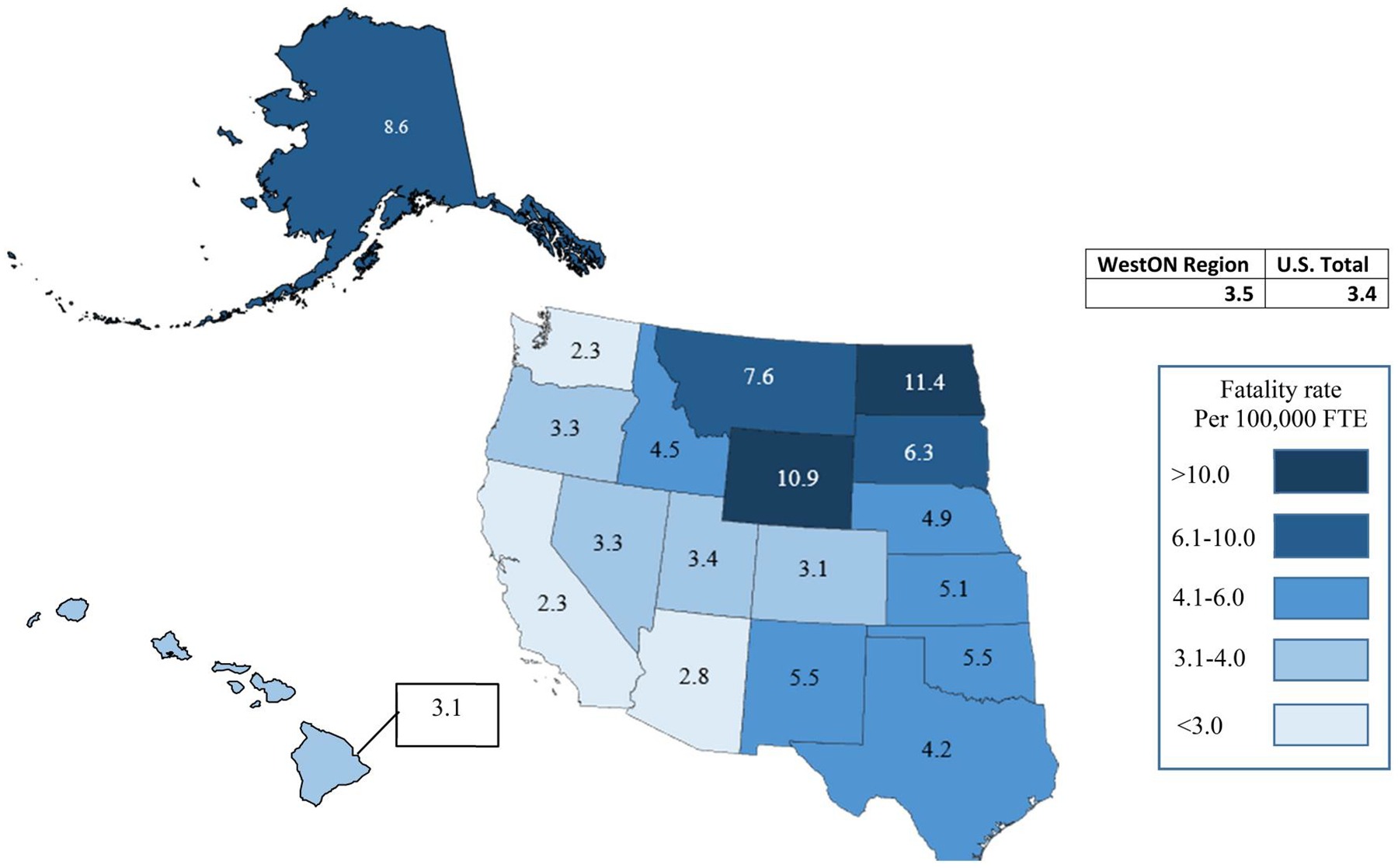
Annual average occupational fatality rates per 100,000 full-time-equivalent workers—WestON states, 2011–2017

From 2011 through 2017, the annual average overall occupational fatality rate for the WestON region was 3.5 fatalities per 100,000 FTE, which was comparable to the annual average overall U.S. fatality rate of 3.4 fatalities per 100,000 FTE during the same period. Three states had fatality rates at least 2.5 times higher than the WestON regional rate: North Dakota, Wyoming, and Alaska (see Table 1 and Figure 1). Four states had fatality rates significantly lower than the regional rate: California, Washington, Arizona, and Colorado (see Table 1). With the exception of Colorado, state overall fatality rates were higher within more inland states compared to more coastal states in the WestON region (Figure 1).

Comparing occupational fatality rates by sex (see Table 1), regionally, male workers had a fatality rate almost 10 times higher than female workers. State fatality rates for male workers were significantly higher than the regional rate for male workers in 11 states (Alaska, Idaho, Kansas, Montana, Nebraska, New Mexico, North Dakota, Oklahoma, South Dakota, Texas, and Wyoming). State rates for female workers were significantly higher than the regional rate for female workers in seven states (Alaska, Montana, Nebraska, North Dakota, Oregon, South Dakota, and Wyoming).

Comparing fatality rates by age groups (see Table 1), regionally, fatality rates increased with each successive age group. Workers aged 65 years and older experienced the highest fatality rate (9.4 fatalities per 100,000 FTE). In North Dakota, for all reported age groupings, state rates were over three times higher than the respective regional rates. Two other states had fatality rates over three times higher than the regional rate for certain age groups: Montana had a rate 3.1 times higher for workers aged 65 years and over, and Wyoming had rates 3.4 and 3.8 times higher for workers aged 25–54 years and 65þ years, respectively.

Compared to the respective WestON regional rates, fatality rates were significantly higher for non-Hispanic White workers in 11 states (Alaska, Idaho, Kansas, Montana, Nebraska, New Mexico, North Dakota, Oklahoma, South Dakota, Texas, and Wyoming), for Hispanic workers in eight states (Alaska, Colorado, Kansas, New Mexico, North Dakota, Oklahoma, Texas, and Wyoming), for non-Hispanic Black workers in three states (Alaska, New Mexico, and North Dakota), and for American Indian/Alaska Native/Asian/Pacific Islander workers (AI/AN/AAPI) in four states (Alaska, Arizona, New Mexico, and Oklahoma; see Table 2). Arizona, North Dakota, and Oklahoma had significantly higher fatality rates than the respective regional rates for multiple racial/ethnic minority groups. Arizona had 2.1 times the rate of fatalities among non-Hispanic AI/AN/AAPI workers and 3.2 times the rate of fatalities among non-Hispanic other or multiple races workers compared to the respective regional rates of those groups. North Dakota had 6.1 times the rate of fatalities among non-Hispanic Black workers and 9.9 times the rate of deaths among Hispanic workers compared to the regional rates for those groups. Oklahoma had 2.3 times the rate of deaths among AI/AN/AAPI workers and 2.4 times the rate of deaths among Hispanic workers compared to the corresponding regional rates. Alaska and New Mexico had significantly higher fatality rates for all racial/ethnic groups compared to respective regional rates.

When comparing fatality rates by foreign-born and U.S.-born status (see Table 2), the states with the three highest occupational fatality rates among foreign-born workers were Wyoming (21.5 fatalities per 100,000 FTE), North Dakota (20.4), and Montana (16.6). States with the highest rates for U.S.-born workers were North Dakota (10.9 fatalities per 100,000 FTE), Wyoming (10.4), and Alaska (8.8). In 10 states (Arizona, California, Colorado, Idaho, Montana, New Mexico, North Dakota, Oklahoma, Texas, and Wyoming), fatality rates for both foreign-born and U.S.-born workers were significantly higher than the respective regional rates. In only one state (Nebraska) was the fatality rate for U.S.-born workers significantly higher than the respective regional rate, while the rate for foreign-born workers was not significantly different from the respective regional rate.

Comparisons within states provided more details on patterns of fatality rates by race/ethnicity and foreign-born/U.S.-born status (see Table 3). In New Mexico, the fatality rate for non-Hispanic Black workers was 2.0 times higher than for non-Hispanic White workers. In Arizona, the fatality rate for non-Hispanic AI/AN/AAPI workers was 1.5 times higher than for non-Hispanic White workers. In Arizona, the fatality rate for non-Hispanic other or multiple race workers was 3.1 times higher than for non-Hispanic White workers; in Hawaii, the rate for non-Hispanic other or multiple race workers was 1.8 times higher than for non-Hispanic White workers. In five states (California, Colorado, Kansas, North Dakota, and Oklahoma), fatality rates for Hispanic workers were significantly higher than for non-Hispanic White workers. Within 10 states (Arizona, California, Colorado, Idaho, Montana, New Mexico, North Dakota, Oklahoma, Texas, and Wyoming), fatality rates were significantly higher among foreign-born workers than among U.S.-born workers.

Comparing regional fatality rates by industry sector group (see Table 4), the three highest fatality rates were in the agriculture, forestry, and fishing industry sector (18.0 fatalities per 100,000 FTE); mining including oil and gas extraction (13.7); and transportation, warehousing, and utilities (11.7). The lowest fatality rate regionally was in the health and social services sector (0.8 fatalities per 100,000 FTE). The highest fatality rate in any industry sector group and state was in agriculture, forestry, and fishing in Alaska (133.1 fatalities per 100,000 FTE). The lowest fatality rate in any state was in health and social services in California (0.6 fatalities per 100,000 FTE).

Comparing industry sector fatality rates within states (see Table 4), the agriculture, forestry, and fishing industry sector had the highest rate of fatalities within twelve states (Alaska, California, Hawaii, Idaho, Kansas, Montana, Nebraska, Oregon, South Dakota, Texas, Utah, and Washington); the mining, oil and gas extraction industry sector had the highest rate of fatalities within three states (Nevada, New Mexico, and North Dakota); and the transportation, warehousing, and utilities industry sector had the highest rate of fatalities within four states (Arizona, Colorado, Oklahoma, and Wyoming). Additionally, North Dakota fatality rates for all reported industry sectors were significantly higher than regional rates.

Comparing fatality rates by event or exposure type (see Table 5), transportation-related incidents were the most frequent event type associated with occupational fatalities for all 19 states (1.6 fatalities per 100,000 FTE). Eleven states (Alaska, Idaho, Kansas, Montana, Nebraska, New Mexico, North Dakota, Oklahoma, South Dakota, Texas, and Wyoming) had significantly higher rates of transportation-related fatalities compared to the regional rate. The highest rate for transportation-related incidents was in North Dakota, which was 4.2 times higher than the regional rate. Falls, slips, and trips were the second most frequent event type associated with occupational fatalities in the WestON region (0.5 fatalities per 100,000 FTE), with seven states (Hawaii, Montana, North Dakota, Oklahoma, South Dakota, Texas, and Wyoming) having significantly higher rates from falls, slips, and trips than the respective regional rate. Two types of events, contact with objects and violence, were each tied as the third most frequent event type associated with occupational fatalities (0.5 fatalities per 100,000 FTE). Twelve states (Idaho, Kansas, Montana, Nebraska, New Mexico, North Dakota, Oklahoma, Oregon, South Dakota, Texas, Utah, and Wyoming) had significantly higher fatality rates associated with contact with objects compared to the regional rate. Four states (Alaska, Nevada, Texas, and Wyoming) had significantly higher rates of violence-related fatalities compared to the corresponding regional rate.

## Discussion

This article summarizes a regional and state-by-state analysis of selected risk factors and their impact on rates of fatal work injuries over a 7-year period in the WestON region. Transportation-related incidents were the most frequent event type associated with occupational fatalities in all 19 WestON states. This is not surprising, given that at the national level transportation events have been the most common cause of occupational fatalities every year since the BLS CFOI began in 1992 (BLS 2011–2017). In this analysis, the transportation-related fatality rate for the WestON region (1.55 per 100,000 FTE) far exceeded the rate for the rest of the United States (0.48 per 100,000 FTE). However, there were large differences among states: 11 states had rates significantly higher than the regional rate, and four states experienced significantly lower rates. Further work is needed to understand modifiable factors associated with elevated transportation-related fatality rates in these states. For example, Hagan-Haynes et al. (2022) performed a cross-sectional survey in North Dakota, Colorado, and Texas, evaluating factors not commonly available in surveillance data such as extended work hours, commuting times, drowsy driving, and safety practices at oil and gas extraction companies.

Concerted effort is needed to address transportation-related risks, since transportation remains a singularly hazardous part of many jobs. By identifying common high-risk worker populations, industries, or work arrangements, multistate collaborations could potentially be leveraged to address this challenge. As an example, a multistate coalition from universities, NIOSH-funded Centers for Agricultural Safety and Health, private corporations, and agricultural organizations worked to address tractor rollovers, a serious hazard in agriculture (Tinc et al. 2016). Among other achievements, this collaboration strengthened a multistate rebate program for tractor rollover protection systems (ROPS), which is active in seven states, saves lives, and has proven cost-effective (National ROPS Rebate Program; Myers et al. 2018). A similar approach might be appropriate for addressing transportation-related fatalities or other hazards.

Consistent with research dating to the early 1980s (Bell et al. 1990; Kisner and Pratt 1997; 1999), this study found higher fatality rates for older workers (ages 65 and older). Our estimate of 9.4 deaths per 100,000 FTE for workers ≥ 65 in the WestON region was slightly lower than the rate of 10.9 for the rest of the United States. However, rates in this group were markedly higher in seven of the 19 WestON states. Prior research has found higher rates of fatalities in older workers associated with machinery (Marsh and Fosbroke 2015), transportation (Myers et al. 2011), falls (Dong et al. 2012), and agricultural work, especially logging (Richey et al. 2023). The association between older workers and deaths in transportation and in the agriculture, forestry, and fishing industry sector has been described repeatedly in the literature, and might be a productive area for future work in the WestON region (Castillo and Malit 1997; Myers et al. 2009; Kachan et al. 2012; Chen et al. 2014; Swanton et al. 2016). For example, a study in Oregon found 55% of occupational fatalities in workers ages 65 years and over were transportation related (Walters et al. 2013). Workers ages 65 years and over had more than a three-fold greater risk for transportation-related fatalities compared to workers of all ages. A study in North Dakota focused on farm-related injuries identified tractor-related incidents as the leading cause of admissions to a regional trauma center, with workers ages 65 years and older accounting for more than half of those incidents (Gilblom et al. 2023). In what the U.S. Census Bureau has called a “gray tsunami,” the entire baby boomer generation will be ages 65 or older by 2030 (U.S. Bureau of Census 2019). As the labor participation rate for this age group is projected to increase in the coming decade (BLS 2022), workers ages 65 and over will soon represent a major segment of the work force. This demographic transition will make it more essential than ever to provide safe working conditions for older workers.

This analysis found elevated occupational fatality rates among several historically marginalized demographic groups, including Black, Hispanic, AI/AN/AAPI, and foreign-born workers. Trends varied substantially by state: rates were higher in at least one of the above-listed groups in 12 of the 19 WestON states compared to White or U.S.-born workers. For foreign-born workers, prior research has not consistently found higher rates of occupational fatalities among foreign-born workers overall, but results have been more consistent for certain subgroups. In 2011, BLS reported 835 deaths in foreign-born workers—16% of total occupational fatalities in the United States in 2008 (BLS 2011). This was roughly proportionate to the representation of foreign-born workers in the labor force (15.6%). However, the same report also showed that in every year over the 17-year period ending in 2008, foreign-born Hispanic/Latino workers died on the job in greater numbers than U.S.-born Hispanic/Latino workers. Two follow-up studies from the BLS produced similar results. From 2006 to 2008, the fatality rate for foreign-born Hispanic/Latino workers (5.7 per 100,000 FTE) exceeded the rate for U.S.-born Hispanic/Latino workers overall (3.6) and for all workers (4.0 per 100,000) (Byler 2013). A more recent study found only a slightly elevated risk for occupational fatalities among foreign-born workers overall (hazard ratio [HR]: 1.15), but higher risk for workers from Central America (HR: 1.5) and Mexico (HR: 1.3) (Byler and Robinson 2018). Further examination of fatalities among historically marginalized workers at the state level by industry, region of birth, or cause of injury could reveal more opportunities for preventing these deaths.

There is an overall scarcity of peer-reviewed occupational safety and health research focusing on American Indian and Alaska Native (AI/AN) workers (Wingate et al. 2023). Some research has documented health risks among AI/AN miners, primarily in coal and uranium mining (Roscoe et al. 1995; Schubauer-Berigan et al. 2009; Hall et al. 2022). A few studies have addressed risks from agricultural work (Goldcamp et al. 2006; Helitzer et al. 2014), animal handling (Duysen et al. 2017), or environmental exposures to AI/AN workers (Klepeis et al. 2016). As part of an effort to increase knowledge and enhance worker safety and health of this population, NIOSH recently published a strategic plan for AI/AN worker safety and health (NIOSH 2023). While NIOSH coordinated the development of the strategic plan, tribes, tribal leaders, tribal-serving organizations, academic researchers, state and local health department staff, and government agencies helped establish the breadth and content of the plan. The plan provides a framework to stimulate collaboration, research, outreach, and partnerships to increase health, safety, and well-being in AI/AN workers.

This study reports occupational fatality rates for AI/AN/AAPI workers as a single group. Collapsing these populations into one group was done to preserve statistical power for comparisons, and because the BLS reporting thresholds would have otherwise required suppression of some data entirely. Unfortunately, this also obscured potential differences between individual groups. As a result, the ability to interpret findings related to individual groups within the AI/AN/AAPI category is limited. It is uncertain the extent to which fatality rates for the AI/AN/AAPI category reflect the risk for more specific groups in that category. Each of these populations likely have distinct work experiences (USDOL 2022), which warrants separate investigations.

### Limitations

This study has several limitations. CFOI data does not capture work-related fatalities caused by illness. Thus, counts related to fatal illnesses acquired on the job were not included (BLS 2012a). Multiple NAICS versions were used to classify industry sectors across the study period, although this is not likely to have altered results meaningfully. Comparisons of fatal work-injury rates among states should be done thoughtfully, as rate differences can be a result of industrial employment patterns and workforce demographics. Additional context (such as is provided in Tables 1–5) should be considered when directly comparing rates. Occupational fatality rates for AI/AN/AAPI workers were analyzed as a single group. Depending on the distribution of workers in the individual groups in this category in a given state and the distribution of fatalities in those groups, rates for the AI/AN/AAPI category could over- or underestimate rates in the groups that comprise the broader category. Foreign-born status can be a proxy for language barriers, access to health resources, health literacy, and other social determinants of health or occupational health disparities (Stanbury and Rosenman 2014). However, these other variables were not available in CFOI data. Thus, foreign-born status as a proxy for these variables has limitations and may be confounded by factors such as region of birth (Byler and Robinson 2018) or age of migration to the United States (Steege et al. 2014). Finally, some of the highest fatality rates occurred in states with relatively small populations. These rates can be highly sensitive to small changes in the numerator and may be unreliable measures for comparing to states with much larger populations.

For this study it was not practical to examine cross-tabulations for each combination of demographic, industry, and event category given the reporting requirements in our data use agreement. However, this study identified several foundational topics for more focused research and prevention efforts. Some of the issues underlined were far more prominent in some states than others, but some cross-cutting issues were common to multiple states, where consultation and sharing of best practices could be helpful.

## Conclusion

Using restricted-access data from BLS CFOI research data files, we examined occupational fatalities in more detail than is available from public BLS CFOI data. We found statistically significant differences in fatal injury patterns across a variety of risk factors in each of the 19 WestON states. This project was intended as a framing study to provide a benchmark for states, researchers, and safety professionals to understand occupational fatalities in the WestON region. The results can provide a starting point for more in-depth research into some of the risk factors highlighted, which varied considerably by state. This report can also help frame programmatic discussions around surveillance, interventions, policies, or collaborations at the state or local level to protect workers.

## Addressing aims and scope

This article is a secondary data analysis that describes and compares patterns of fatal work-related injuries by demographics and employment. The purpose of this article is to enhance the knowledge of, and encourage collaboration among, occupational health scientists to address work-related fatality events and exposure risks in different demographic populations, geographic locations, industries, and occupations.

## Figures and Tables

**Table 1.** Annual average state occupational fatality rates* compared to annual average WestON region occupational fatality rates, select demographics, 2011–2017, Census of Fatal Occupational Injuries

			Sex								Age Group					
			Male		Female		15–19		20–24		25–54		55–64		65+	
	Average Annual Rate	RR (95% CIs)	Rate	RR (95% CIs)	Rate	RR (95% CIs)	Rate	RR (95% CIs)	Rate	RR (95% CIs)	Rate	RR (95% CIs)	Rate	RR (95% CIs)	Rate	RR (95% CIs)
U.S. Total	3.42	N/A	5.58	N/A	0.59	N/A	2.29	N/A	2.40	N/A	2.90	N/A	4.34	N/A	10.38	N/A
WestON Region	3.49	N/A	5.60	N/A	0.58	N/A	2.15	N/A	2.69	N/A	2.99	N/A	4.60	N/A	9.43	N/A
Rest of U.S.	3.37	N/A	5.57	N/A	0.60	N/A	2.37	N/A	2.23	N/A	2.85	N/A	4.21	N/A	10.89	N/A
**WestON States**																
Alaska	**8.56** ^†^	**2.45 (2.13, 2.83)**	**13.78**	**2.46 (2.12, 2.85)**	**1.56**	**2.67 (1.60, 4.46)**	–^‡^	–	7.00		**7.37**	**2.46 (2.04, 2.97)**	**10.81**	**2.35 (1.73, 3.20)**	–	–
Arizona	2.77	0.79 (0.73, 0.86)	4.50	0.80 (0.73, 0.88)	0.47	0.80 (0.58, 1.10)	–	–	–	–	2.46	0.82 (0.74, 0.92)	3.66	0.80 (0.66, 0.96)	6.65	0.71 (0.54, 0.93)
California	2.26	0.65 (0.62, 0.67)	3.58	0.64 (0.61, 0.67)	0.43	0.73 (0.63, 0.86)	–	–	1.48		1.99	0.67 (0.63, 0.70)	2.99	0.65 (0.59, 0.71)	5.69	0.60 (0.53, 0.68)
Colorado	3.07	0.88 (0.81, 0.96)	4.85	0.87 (0.79, 0.95)	0.59	1.01 (0.75, 1.37)	–	–	–	–	2.55	0.85 (0.76, 0.95)	4.58	1.00 (0.84, 1.19)	7.62	0.81 (0.62, 1.06)
Hawaii	3.06	0.88 (0.74, 1.04)	5.31	0.95 (0.79, 1.14)	0.37	0.64 (0.30, 1.34)	–	–	–	–	2.71	0.90 (0.72, 1.14)	3.41	0.74 (0.51, 1.08)	7.42	0.79 (0.49, 1.25)
Idaho	**4.48**	**1.28 (1.12, 1.47)**	**7.11**	**1.27 (1.11, 1.46)**	0.56	0.96 (0.53, 1.74)	–	–	4.10		3.09	**1**.03 (0.85, **1**.25)	**6.39**	**1.39 (1.05, 1.83)**	**18.52**	**1.96 (1.42, 2.71)**
Kansas	**5.05**	**1.45 (1.32, 1.58)**	**8.42**	**1.50 (1.37, 1.65)**	0.57	0.97 (0.64, 1.47)	–	–	3.80		**4.11**	**1.37 (1.21, 1.56)**	5.18	1.13 (0.91, 1.39)	**19.83**	**2.10 (1.72, 2.58)**
Montana	**7.63**	**2.18 (1.92, 2.48)**	**11.93**	**2.13 (1.86, 2.44)**	**1.98**	**3.41 (2.32, 5.00)**	–	–	–	–	**5.89**	**1.97 (1.64, 2.36)**	**8.21**	**1.79 (1.35, 2.36)**	**29.36**	**3.11 (2.40, 4.05)**
Nebraska	**4.93**	**1.41 (1.26, 1.58)**	**7.84**	**1.40 (1.25, 1.57)**	**1.12**	**1.92 (1.35, 2.74)**	–	–	–	–	3.15	**1**.05 (0.89, **1**.25)	**6.93**	**1.51 (1.21, 1.89)**	**25.25**	**2.68 (2.16, 3.32)**
Nevada	3.27	0.94 (0.83, 1.05)	5.19	0.93 (0.82, 1.05)	0.69	1.18 (0.79, 1.75)	–	–	1.02		3.08	1.03 (0.89, 1.19)	5.00	1.09 (0.85, 1.39)	–	–
New Mexico	**5.47**	**1.57 (1.40, 1.75)**	**9.29**	**1.66 (1.48, 1.86)**	0.52	0.90 (0.52, 1.56)	–	–	6.50		**4.94**	**1.65 (1.43, 1.91)**	**6.23**	**1.35 (1.05, 1.74)**	–	–
North Dakota	**11.37**	**3.26 (2.91, 3.64)**	**18.45**	**3.30 (2.94, 3.70)**	**1.07**	**1.84 (1.04, 3.26)**	–	–	–	–	**9.82**	**3.28 (2.82, 3.81)**	**14.06**	**3.06 (2.41, 3.88)**	**29.94**	**3.18 (2.30, 4.38)**
Oklahoma	**5.46**	**1.56 (1.44, 1.69)**	**8.79**	**1.57 (1.45, 1.70)**	0.77	1.32 (0.96, 1.83)	–	–	–	–	**5.25**	**1.75 (1.59, 1.94)**	**5.70**	**1.24 (1.03, 1.50)**	8.87	0.94 (0.73, 1.22)
Oregon	3.31	0.95 (0.86, 1.05)	5.23	0.94 (0.84, 1.04)	**0.82**	**1.41 (1.04, 1.92)**	–	–	–	–	2.64	0.88 (0.77, 1.01)	4.60	1.00 (0.82, 1.23)	10.62	1.13 (0.87, 1.45)
South Dakota	**6.34**	**1.82 (1.57, 2.10)**	**10.09**	**1.80 (1.55, 2.10)**	**1.21**	**2.07 (1.24, 3.45)**	–	–	–	–	**4.45**	**1.49 (1.20, 1.85)**	**8.23**	**1.79 (1.34, 2.40)**	**22.20**	**2.36 (1.73, 3.21)**
Texas	**4.24**	**1.21 (1.17, 1.26)**	**6.81**	**1.22 (1.17, 1.26)**	0.59	1.01 (0.87, 1.18)	–	–	3.57		**3.73**	**1.25 (1.19, 1.31)**	**5.80**	**1.26 (1.16, 1.37)**	9.45	1.00 (0.89, 1.13)
Utah	3.35	0.96 (0.85, 1.08)	4.95	0.89 (0.79, 1.00)	0.74	1.27 (0.85, 1.89)	–	–	–	–	3.19	**1**.07 (0.92, **1**.23)	4.52	0.98 (0.75, 1.28)	10.46	**1.11** (0.78, **1**.57)
Washington	2.27	0.65 (0.59, 0.71)	3.63	0.65 (0.59, 0.71)	0.44	0.75 (0.55, 1.04)	–	–	–	–	1.78	0.60 (0.53, 0.67)	3.24	0.70 (0.59, 0.84)	8.18	0.87 (0.69, 1.10)
Wyoming	**10.91**	**3.12 (2.73, 3.58)**	**16.61**	**2.97 (2.58, 3.41)**	**2.18**	**3.74 (2.31, 6.04)**	–	–	–	–	**9.75**	**3.26 (2.73, 3.89)**	**10.74**	**2.34 (1.70, 3.21)**	**35.91**	**3.81 (2.74, 5.30)**

* Per 100,000 full-time equivalent (FTE) workers ages ≥15 years. Rates were calculated by NIOSH based on the number of fatalities from restricted data from the Bureau of Labor Statistics (BLS) Census of Fatal Occupational Injuries from 2011 to 2017; hours worked based on full-time-equivalent workers from the BLS Current Population Survey, 2011–2017. The views expressed here do not necessarily reflect the views of the BLS.

† Rates in bold were significantly higher than the WestON regional rate (*p*<0.05).

‡ Dashes indicate that data did not meet BLS publication criteria.

**Table 2.** Annual average state occupational fatality rates* compared to annual average WestON region occupational fatality rates, select demographics, 2011–2017, Census of Fatal Occupational Injuries.

					Race/Ethnicity						Foreign-born			
	White, Non-Hispanic		Black, Non-Hispanic		American Indian/Alaska Native/Asian/Pacific Islanders		Other Race, Non-Hispanic		Hispanic		Foreign-born		US-born	
	Rate	RR (95% CIs)	Rate	RR (95% CIs)	Rate	RR (95% CIs)	Rate	RR (95% CIs)	Rate	RR (95% CIs)	Rate	RR (95% CIs)	Rate	RR (95% CIs)
U.S. Total	3.53	N/A	3.25	N/A	1.91	N/A	2.62	N/A	3.72	N/A	3.57	N/A	3.38	N/A
WestON Region	3.73	N/A	3.22	N/A	1.86	N/A	2.50	N/A	3.72	N/A	3.68	N/A	3.44	N/A
Rest of U.S.	3.45	N/A	3.26	N/A	1.97	N/A	2.73	N/A	3.73	N/A	3.48	N/A	3.36	N/A
**WestON States**														
Alaska	**8.01** ^†^	**2.15 (1.80, 2.56)**	**9.72**	**3.02 (1.43, 6.36)**	**7.85**	**4.22 (2.93, 6.09)**	**13.45**	**5.38 (3.16, 9.14)**	**11.96**	**3.22 (2.00, 5.18)**	**6.94**	**1.89 (1.19, 3.00)**	**8.77**	**2.55 (2.20 2.96)**
Arizona	2.58	0.69 (0.61, 0.78)	3.15	0.98 (0.66, 1.45)	**3.96**	**2.13 (1.58, 2.86)**	**8.04**	**3.21 (1.92, 5.38)**	2.65	0.71 (0.61, 0.83)	3.35	0.91 (0.77, 1.09)	2.61	0.76 (0.69, 0.84)
California	2.28	0.61 (0.57, 0.65)	2.27	0.71 (0.58, 0.86)	1.27	0.68 (0.59, 0.80)	0.64	0.26 (0.14, 0.47)	2.74	0.74 (0.69, 0.79)	2.80	0.76 (0.71, 0.82)	1.97	0.57 (0.54, 0.61)
Colorado	2.77	0.74 (0.67, 0.82)	2.36	0.73 (0.44, 1.22)	–^‡^	–	–	–	**4.74**	**1.27 (1.08, 1.50)**	4.32	1.17 (0.96, 1.44)	2.89	0.84 (0.76, 0.92)
Hawaii	3.85	1.03 (0.72, 1.47)	–	–	1.85	1.00 (0.73, 1.36)	6.90	2.76 (1.96, 3.89)	–	–	3.93	1.07 (0.78, 1.47)	2.79	0.81 (0.66, 1.00)
Idaho	**4.45**	**1.19 (1.03, 1.38)**	–	–	–	–	–	–	5.18	1.39 (0.99, 1.96)	**6.51**	**1.77 (1.27, 2.47)**	**4.23**	**1.23 (1.06, 1.42)**
Kansas	**5.07**	**1.36 (1.22, 1.50)**	4.03	1.25 (0.78, 2.00)	–	–	–	–	**7.15**	**1.92 (1.50, 2.46)**	**5.29**	**1.44 (1.09, 1.90)**	**5.02**	**1.46 (1.33, 1.61)**
Montana	**7.83**	**2.10 (1.84, 2.40)**	–	–	4.16	2.24 (0.93, 5.39)	–	–	–	–	**16.59**	**4.51 (2.76, 7.38)**	**7.34**	**2.14 (1.87, 2.44)**
Nebraska	**5.03**	**1.35 (1.19, 1.52)**	4.92	1.53 (0.84, 2.77)	–	–	–	–	4.57	1.23 (0.85, 1.77)	4.73	1.29 (0.91, 1.82)	**4.95**	**1.44 (1.28, 1.62)**
Nevada	3.67	0.98 (0.84, 1.14)	2.32	0.72 (0.42, 1.23)	2.22	1.19 (0.76, 1.86)	3.20	1.28 (0.53, 3.12)	3.12	0.84 (0.66, 1.06)	3.44	0.94 (0.75, 1.17)	3.20	0.93 (0.81, 1.07)
New Mexico	**5.84**	**1.56 (1.33, 1.85)**	**11.64**	**3.62 (2.04, 6.40)**	**3.84**	**2.06 (1.36, 3.13)**	**11.21**	**4.48 (1.84, 10.92)**	**5.13**	**1.38 (1.15, 1.65)**	**7.23**	**1.97 (1.52, 2.55)**	**5.18**	**1.51 (1.33, 1.71)**
North Dakota	**10.80**	**2.89 (2.56, 3.27)**	**19.78**	**6.14 (3.18, 11.86)**	–	–	–	–	**36.81**	**9.90 (6.68, 14.67)**	**20.35**	**5.54 (3.76, 8.14)**	**10.93**	**3.18 (2.83, 3.58)**
Oklahoma	**5.60**	**1.50 (1.36, 1.65)**	4.54	1.41 (1.00, 1.99)	**4.20**	**2.26 (1.67, 3.06)**	1.68	0.67 (0.37, 1.21)	**8.82**	**2.37 (1.93, 2.91)**	**8.91**	**2.42 (1.97, 2.98)**	**5.12**	**1.49 (1.37, 1.62)**
Oregon	3.51	0.94 (0.84, 1.05)	–	–	0.97	0.52 (0.25, 1.09)	–	–	3.65	0.98 (0.74, 1.31)	2.98	0.81 (0.61, 1.07)	3.36	0.98 (0.88, 1.09)
South Dakota	**6.56**	**1.76 (1.51, 2.04)**	–	–	3.07	1.65 (0.69, 3.98)	–	–	–	–	**7.67**	**2.09 (1.08, 4.02)**	**6.29**	**1.83 (1.58, 2.12)**
Texas	**4.38**	**1.17 (1.11, 1.24)**	3.63	1.13 (0.99, 1.29)	2.09	1.12 (0.90, 1.40)	1.45	0.58 (0.31, 1.07)	**4.63**	**1.25 (1.17, 1.32)**	**4.88**	**1.33 (1.23, 1.43)**	**4.05**	**1.18 (1.13, 1.23)**
Utah	3.31	0.89 (0.78, 1.01)	–	–	3.12	1.67 (0.90, 3.13)	–	–	3.67	0.99 (0.72, 1.34)	4.06	1.10 (0.82, 1.49)	3.25	0.95 (0.83, 1.07)
Washington	2.41	0.65 (0.58, 0.72)	1.18	0.37 (0.18, 0.74)	1.39	0.75 (0.53, 1.07)	1.17	0.47 (0.23, 0.96)	2.78	0.75 (0.58, 0.95)	2.09	0.57 (0.46, 0.71)	2.31	0.67 (0.61, 0.74)
Wyoming	**10.91**	**2.92 (2.53, 3.38)**	–	–	–	–	–	–	**11.93**	**3.21 (2.04, 5.03)**	**21.52**	**5.85 (3.77, 9.10)**	**10.39**	**3.02 (2.62, 3.48)**

* Per 100,000 full-time-equivalent (FTE) workers ages ≥ 15 years. Rates were calculated by NIOSH based on the number of fatalities from restricted data from the Bureau of Labor Statistics (BLS) Census of Fatal Occupational Injuries from 2011 to 2017; hours worked based on full-time-equivalent workers from the BLS Current Population Survey, 2011–2017. The views expressed here do not necessarily reflect the views of the BLS.

† Rates in bold were significantly higher than the WestON regional rate (*p*<0.05).

‡ Dashes indicate that data did not meet BLS publication criteria.

**Table 3.** Comparison of annual average occupational fatality rates* within state, select demographics, 2011–2017, Census of Fatal Occupational Injuries.

					Race/Ethnicity						Foreign-born			
	White, Non-Hispanic		Black, Non-Hispanic		American Indian/Alaska Native/Asian/Pacific Islanders		Other Race, Non-Hispanic		Hispanic		Foreign-born		US-born	
WestON States	Rate	RR (95% CIs)	Rate	RR (95% CIs)	Rate	RR (95% CIs)	Rate	RR (95% CIs)	Rate	RR (95% CIs)	Rate	RR (95% CIs)	Rate	RR (95% CIs)
Alaska	8.01	N/A	9.72	1.21 (0.57, 2.60)	7.85	0.98 (0.66, 1.46)	13.45	1.68 (0.98, 2.87)	11.96	1.49 (0.90, 2.48)	6.94	0.79 (0.49, 1.29)	8.77	N/A
Arizona	2.58	N/A	3.15	1.22 (0.82, 1.83)	**3.96** ^‡^	**1.53 (1.13, 2.09)**	**8.04**	**3.12 (1.88, 5.15)**	2.65	1.03 (0.85, 1.25)	**3.35** ^‡^	**1.28 (1.05, 1.56)**	2.61	N/A
California	2.28	N/A	2.27	1.00 (0.83, 1.20)	1.27	0.56 (0.48, 0.65)	0.64	0.28 (0.15, 0.51)	2.74	1.20 (1.11, 1.31)	**2.80**	**1.42 (1.31, 1.53)**	1.97	N/A
Colorado	2.77	N/A	2.36	0.73 (0.44, 1.22)	–**	–	–	–	4.74	1.71 (1.41, 2.07)	**4.32**	**1.49 (1.20, 1.86)**	2.89	N/A
Hawaii	3.85	N/A	–	–	1.85	0.48 (0.30, 0.76)	**6.90**	**1.80 (1.13, 2.86)**	-	-	3.93	1.41 (0.97, 2.06)	2.79	N/A
Idaho	4.45	N/A	–	–	–	–	–	–	5.18	1.16 (0.80, 1.69)	**6.51**	**1.54 (1.07, 2.21)**	4.23	N/A
Kansas	5.07	N/A	4.03	0.80 (0.50, 1.28)	–	–	–	–	7.15	1.41 (1.08, 1.84)	5.29	1.05 (0.79, 1.41)	5.02	N/A
Montana	7.83	N/A	–	–	4.16	0.53 (0.22, 1.29)	–	–	–	–	**16.59**	**2.26 (1.36, 3.75)**	7.34	N/A
Nebraska	5.03	N/A	4.92	0.98 (0.54, 1.79)	–	–	–	–	4.57	0.91 (0.62, 1.33)	4.73	0.96 (0.66, 1.38)	4.95	N/A
Nevada	3.67	N/A	2.32	0.63 (0.37, 1.09)	2.22	0.60 (0.38, 0.96)	3.20	0.87 (0.36, 2.13)	3.12	0.85 (0.64, 1.12)	3.44	1.08 (0.83, 1.40)	3.20	N/A
New Mexico	5.84	N/A	11.64	1.99 (1.11, 3.59)	3.84	0.66 (0.42, 1.02)	11.21	1.92 (0.79, 4.68)	5.13	0.88 (0.69, 1.11)	**7.23**	**1.40 (1.05, 1.86)**	5.18	N/A
North Dakota	10.80	N/A	19.78	1.83 (0.94, 3.56)	–	–	–	–	**36.81**	**3.41 (2.26, 5.14)**	**20.35**	**1.86 (1.25, 2.78)**	10.93	N/A
Oklahoma	5.60	N/A	4.54	0.81 (0.57, 1.15)	4.20	0.75 (0.55, 1.02)	1.68	0.30 (0.17, 0.53)	**8.82**	**1.57 (1.26, 1.97)**	**8.91**	**1.74 (1.40, 2.16)**	5.12	N/A
Oregon	3.51	N/A	–	–	0.97	0.27 (0.13, 0.58)	–	–	3.65	1.04 (0.77, 1.41)	2.98	0.89 (0.66, 1.19)	3.36	N/A
South Dakota	6.56	N/A	–	–	3.07	0.47 (0.19, 1.14)	–	–	–	–	7.67	1.22 (0.62, 2.38)	6.29	N/A
Texas	4.38	N/A	3.63	0.83 (0.74, 0.93)	2.09	0.48 (0.39, .059)	1.45	0.33 (0.18, 0.60)	4.63	1.06 (0.98, 1.13)	**4.88**	**1.21 (1.12, 1.30)**	4.05	N/A
Utah	3.31	N/A	–	–	3.12	0.94 (0.50, 1.77)	–	–	3.67	1.11 (0.79, 1.54)	4.06	1.25 (0.91, 1.72)	3.25	N/A
Washington	2.41	N/A	1.18	0.49 (0.24, 0.99)	1.39	0.58 (0.40, 0.83)	1.17	0.49 (0.24, 0.98)	2.78	1.15 (0.88, 1.50)	2.09	0.91 (0.72, 1.15)	2.31	N/A
Wyoming	10.91	N/A	–	–	–	–	–	–	11.93	1.09 (0.68, 1.75)	**21.52**	**2.07 (1.31, 3.28)**	10.39	N/A

* Per 100,000 full-time-equivalent (FTE) workers ages ≥ 15 years. Rates were calculated by NIOSH based on the number of fatalities from restricted data from the Bureau of Labor Statistics (BLS) Census of Fatal Occupational Injuries from 2011 to 2017; hours worked based on full-time-equivalent workers from the BLS Current Population Survey, 2011–2017. The views expressed here do not necessarily reflect the views of the BLS.

† Rates by race/ethnicity that are bolded were significantly higher than the corresponding rate for Non-Hispanic Whites (*p*<0.05).

‡ Foreign-born rates that are bolded were significantly higher than the corresponding rate for U.S.-born workers (*p*<0.05).

** Dashes indicate that data did not meet BLS publication criteria

**Table 4.** Annual average state occupational fatality rates* compared to annual average WestON region occupational fatality rates, industry sector^†^, 2011–2017, Census of Fatal Occupational Injuries.

	Ag/For/Fish		Construction		Health & Social Services		Manufacturing		Mining, inc Oil/Gas		Services, exc health		Trade		Trans/Warehouse/Utilities	
	Rate	RR (95% CIs)	Rate	RR (95% CIs)	Rate	RR (95% CIs)	Rate	RR (95% CIs)	Rate	RR (95% CIs)	Rate	RR (95% CIs)	Rate	RR (95% CIs)	Rate	RR (95% CIs)
U.S. Total	23.02	N/A	9.55	N/A	0.74	N/A	2.05	N/A	12.92	N/A	2.08	N/A	2.48	N/A	10.93	N/A
WestON Region	18.03	N/A	8.86	N/A	0.78	N/A	2.04	N/A	13.69	N/A	2.00	N/A	2.26	N/A	11.67	N/A
Rest of U.S.	27.26	N/A	9.96	N/A	0.73	N/A	2.06	N/A	11.28	N/A	2.13	N/A	2.60	N/A	10.53	N/A
**WestON States**																
Alaska	**133.14** ^‡^	**7.38 (5.84, 9.34)**	9.50	1.07 (0.67, 1.73)	–**	–	–	–	6.50	0.47 (0.21, 1.06)	**3.92**	**1.96 (1.45, 2.66)**	–	–	**21.01**	**1.80 (1.32, 2.45)**
Arizona	10.81	0.60 (0.41, 0.88)	6.15	0.69 (0.56, 0.86)	0.66	0.85 (0.52, 1.41)	1.41	0.69 (0.45, 1.06)	5.35	0.39 (0.16, 0.94)	2.10	1.05 (0.91, 1.21)	1.78	0.78 (0.59, 1.04)	11.88	1.02 (0.85, 1.22)
California	10.78	0.60 (0.52, 0.68)	5.96	0.67 (0.61, 0.75)	0.61	0.79 (0.62, 1.00)	1.48	0.73 (0.62, 0.86)	4.91	0.36 (0.21, 0.60)	1.52	0.76 (0.70, 0.82)	1.81	0.80 (0.70, 0.91)	7.28	0.62 (0.56, 0.69)
Colorado	9.76	0.54 (0.39, 0.76)	8.12	0.92, 0.76, 1.10)	0.82	1.06 (0.65, 1.72)	1.57	0.77 (0.51, 1.18)	10.76	0.79 (0.56, 1.11)	1.81	0.91 (0.78, 1.06)	1.91	0.84 (0.62, 1.15)	12.74	1.09 (0.90, 1.32)
Hawaii	15.92	0.88 (0.47, 1.64)	9.98	1.13 (0.80, 1.59)	–	–	–	–	–	–	2.43	1.22 (0.93, 1.58)	–	–	4.32	0.37 (0.21, 0.65)
Idaho	21.59	1.20 (0.94, 1.52)	8.54	0.96 (0.68, 1.37)	0.86	1.10 (0.46, 2.67)	1.52	0.75 (0.39, 1.44)	20.63	1.51 (0.68, 3.37)	1.88	0.94 (0.68, 1.29)	2.82	1.25 (0.79, 1.96)	13.59	1.17 (0.85, 1.61)
Kansas	**36.81**	**2.04 (1.70, 2.46)**	10.78	1.22 (0.96, 1.55)	0.87	1.12 (0.61, 2.04)	**3.42**	**1.68 (1.24, 2.27)**	**27.12**	**1.98 (1.33, 2.95)**	1.88	0.94 (0.75, 1.17)	**3.19**	**1.41 (1.02, 1.95)**	**20.89**	**1.79 (1.45, 2.21)**
Montana	**38.70**	**2.15 (1.73, 2.66)**	**13.85**	**1.56 (1.13, 2.16)**	–	–	**6.12**	**3.00 (1.61, 5.60)**	20.24	1.48 (0.79, 2.76)	**3.67**	**1.84 (1.40, 2.42)**	–	–	**18.52**	**1.59 (1.11, 2.28)**
Nebraska	**27.18**	**1.51 (1.24, 1.83)**	9.14	1.03 (0.77, 1.39)	–	–	2.12	1.04 (0.65, 1.66)	–	–	2.38	1.19 (0.93, 1.52)	**3.33**	**1.47 (1.03, 2.10)**	13.30	1.14 (0.85, 1.52)
Nevada	8.99	0.50 (0.22, 1.11)	6.38	0.72 (0.52, 1.00)	1.31	1.69 (0.90, 3.17)	3.11	1.53 (0.88, 2.64)	14.35	1.05 (0.66, 1.67)	**2.56**	**1.28 (1.07, 1.53)**	1.15	0.51 (0.30, 0.86)	11.32	0.97 (0.74, 1.26)
New Mexico	10.07	0.56 (0.34, 0.91)	**13.41**	**1.51 (1.16, 1.97)**	0.92	1.19 (0.56, 2.52)	5.49	2.69 (1.64, 4.42)	**35.06**	**2.56 (1.94, 3.38)**	2.45	1.22 (0.97, 1.55)	2.99	1.32 (0.86, 2.03)	**26.78**	**2.29 (1.80, 2.92)**
North Dakota	**24.57**	**1.36 (1.05, 1.76)**	**32.83**	**3.71 (2.92, 4.70)**	–	–	–	–	**54.64**	**3.99 (3.12, 5.11)**	**3.71**	**1.86 (1.35, 2.56)**	**5.13**	**2.27 (1.47, 3.49)**	**30.29**	**2.60 (1.93, 3.50)**
Oklahoma	11.56	0.64 (0.47, 0.88)	**15.94**	**1.80 (1.51, 2.14)**	1.20	1.54 (0.96, 2.48)	3.56	1.75 (1.27, 2.39)	12.34	0.90 (0.71, 1.14)	**2.61**	**1.31 (1.10, 1.56)**	**3.26**	**1.44 (1.10, 1.89)**	**21.28**	**1.82 (1.55, 2.15)**
Oregon	**25.30**	**1.40 (1.16, 1.69)**	7.09	0.80 (0.61, 1.05)	–	–	–	–	–	–	1.77	0.89 (0.72, 1.09)	1.77	0.78 (0.54, 1.13)	12.32	1.06 (0.84, 1.34)
South Dakota	22.93	1.27 (0.99, 1.64)	**19.29**	**2.18 (1.61, 2.96)**	–	–	–	–	–	–	2.69	1.35 (0.94, 1.93)	**4.95**	**2.19 (1.40, 3.40)**	12.18	1.04 (0.65, 1.68)
Texas	16.57	0.92, 0.78, 1.09)	**10.95**	**1.24 (1.14, 1.34)**	0.77	0.99 (0.77, 1.28)	**2.65**	**1.30 (1.12, 1.52)**	12.79	0.93 (0.82, 1.06)	**2.46**	**1.23 (1.15, 1.32)**	**2.79**	**1.23 (1.09, 1.40)**	**13.16**	**1.13 (1.04, 1.23)**
Utah	16.69	0.93 (0.58, 1.47)	7.55	0.85 (0.65, 1.11)	**2.01**	**2.59 (1.59, 4.21)**	2.04	1.00 (0.66, 1.52)	8.34	0.61 (0.38, 0.99)	2.00	1.00 (0.81, 1.25)	1.82	0.81 (0.53, 1.22)	13.80	1.18 (0.92, 1.53)
Washington	15.00	0.83 (0.68, 1.02)	5.55	0.63 (0.50, 0.78)	–	–	1.36	0.67 (0.48, 0.93)	–	–	1.23	0.61 (0.51, 0.74)	1.71	0.75 (0.57, 0.99)	6.54	0.56 (0.45, 0.70)
Wyoming	**33.93**	**1.88 (1.35, 2.63)**	13.24	1.49 (0.97, 2.30)	–	–	–	–	10.82	0.79 (0.55, 1.15)	**6.75**	**3.38 (2.57, 4.44)**	**5.53**	**2.44 (1.41, 4.22)**	**38.43**	**3.29 (2.51, 4.31)**

* Per 100,000 full-time-equivalent (FTE) workers ages ≥ 15 years. Rates were calculated by NIOSH based on the number of fatalities from restricted data from the Bureau of Labor Statistics (BLS) Census of Fatal Occupational Injuries from 2011 to 2017; hours worked based on full-time-equivalent workers from the BLS Current Population Survey, 2011–2017. The views expressed here do not necessarily reflect the views of the BLS.

† Industry in which the decedent worked was coded according to the 2002 North American Industry Classification System (NAICS). The detailed codes from the 20 NAICS sectors were combined into eight industry sectors according to the similarity of their occupational safety and health risks.

‡ Rates in bold were significantly higher than the WestON regional rate (*p*<0.05).

** Dashes indicate that data did not meet BLS publication criteria

**Table 5.** Annual average state occupational fatality rates* compared to annual average WestON region occupational fatality rates, select event/exposure groups^†^, 2011–2017, Census of Fatal Occupational Injuries

	Violence		Transportation		Fires & Explosions		Falls/Slips/Trips		Exposures		Contact with Objects	
	Rate	RR (95% CIs)	Rate	RR (95% CIs)	Rate	RR (95% CIs)	Rate	RR (95% CIs)	Rate	RR (95% CIs)	Rate	RR (95% CIs)
U.S. Total	0.37	N/A	0.85	N/A	0.05	N/A	0.37	N/A	0.21	N/A	0.34	N/A
WestON Region	0.51	N/A	1.55	N/A	0.10	N/A	0.54	N/A	0.27	N/A	0.51	N/A
Rest of U.S.	0.29	N/A	0.48	N/A	0.02	N/A	0.28	N/A	0.17	N/A	0.24	N/A
**WestON States**												
Alaska	**1.33** ^‡^	**2.59 (1.81, 3.72)**	**5.01**	**3.24 (2.69, 3.90)**	–**	–	0.62	1.16 (0.68, 1.95)	**0.62**	**2.34 (1.38, 3.96)**	–	–
Arizona	0.59	1.15 (0.95, 1.39)	1.06	0.68 (0.60, 0.79)	–	–	0.40	0.75 (0.59, 0.94)	0.29	1.10 (0.84, 1.44)	0.26	0.51 (0.39, 0.68)
California	0.47	0.91 (0.83, 1.00)	0.81	0.52 (0.49, 0.56)	0.04	0.40 (0.29, 0.54)	0.42	0.79 (0.71, 0.87)	0.19	0.71 (0.61, 0.83)	0.32	0.62 (0.55, 0.69)
Colorado	0.48	0.93 (0.75, 1.16)	1.43	0.93 (0.82, 1.05)	–	–	0.51	0.95 (0.77, 1.18)	0.18	0.67 (0.47, 0.96)	0.38	0.74 (0.58, 0.95)
Hawaii	0.39	0.75 (0.46, 1.23)	1.06	0.69 (0.51, 0.92)	–	–	**0.89**	**1.66 (1.20, 2.30)**	0.27	1.00 (0.55, 1.81)	–	–
Idaho	0.29	0.56 (0.33, 0.94)	**2.54**	**1.64 (1.37, 1.96)**	–	–	–	–	0.22	0.85 (0.47, 1.54)	**0.86**	**1.69 (1.24, 2.29)**
Kansas	0.58	1.13 (0.86, 1.48)	**2.76**	**1.79 (1.58, 2.02)**	–	–	0.49	0.90 (0.67, 1.21)	0.27	1.03 (0.70, 1.53)	**0.70**	**1.37 (1.07, 1.75)**
Montana	–	–	**3.27**	**2.12 (1.74, 2.57)**	**0.22**	**2.14 (1.01, 4.53)**	**1.27**	**2.37 (1.73, 3.24)**	0.22	0.84 (0.40, 1.76)	**1.59**	**3.12 (2.36, 4.13)**
Nebraska	0.39	0.77 (0.52, 1.13)	**2.67**	**1.73 (1.48, 2.01)**	0.18	1.75 (0.99, 3.12)	0.55	1.02 (0.73, 1.41)	–	–	**0.92**	**1.82 (1.41, 2.34)**
Nevada	**0.69**	**1.35 (1.04, 1.75)**	1.30	0.84 (0.70, 1.02)	0.02	0.23 (0.06, 0.91)	0.52	0.96 (0.71, 1.30)	–	–	0.35	0.69 (0.48, 0.99)
New Mexico	0.56	1.10 (0.77, 1.55)	**3.20**	**2.07 (1.78, 2.40)**	**0.21**	**2.03 (1.14, 3.61)**	–	–	0.30	1.13 (0.70, 1.82)	**0.79**	**1.55 (1.16, 2.09)**
North Dakota	–	–	**6.45**	**4.17 (3.59, 4.84)**	**0.62**	**5.97 (3.67, 9.71)**	**0.95**	**1.76 (1.20, 2.60)**	0.44	1.65 (0.93, 2.91)	**2.26**	**4.44 (3.44, 5.72)**
Oklahoma	0.43	0.84 (0.63, 1.11)	**3.00**	**1.94 (1.74, 2.16)**	–	–	**0.69**	**1.29 (1.03, 1.61)**	**0.47**	**1.78 (1.36, 2.33)**	**0.70**	**1.38 (1.10, 1.71)**
Oregon	0.49	0.96 (0.74, 1.25)	1.42	0.92 (0.79, 1.07)	–	–	0.42	0.78 (0.59, 1.03)	0.16	0.60 (0.38, 0.94)	**0.67**	**1.31 (1.05, 1.64)**
South Dakota	0.58	1.12 (0.70, 1.81)	**2.85**	**1.84 (1.48, 2.29)**	–	–	**1.15**	**2.15 (1.53, 3.01)**	–	–	**1.05**	**2.06 (1.45, 2.95)**
Texas	**0.57**	**1.11 (1.01, 1.23)**	**1.89**	**1.22 (1.16, 1.29)**	**0.15**	**1.46 (1.20, 1.79)**	**0.65**	**1.21 (1.10, 1.33)**	**0.37**	**1.38 (1.21, 1.57)**	**0.60**	**1.18 (1.07, 1.30)**
Utah	0.48	0.94 (0.70, 1.28)	1.48	0.95 (0.80, 1.14)	–	–	0.36	0.67 (0.47, 0.95)	–	–	**0.70**	**1.37 (1.07, 1.77)**
Washington	0.31	0.60 (0.47, 0.77)	0.83	0.54 (0.46, 0.62)	–	–	0.56	1.05 (0.87, 1.26)	–	–	0.37	0.73 (0.59, 0.92)
Wyoming	**1.36**	**2.66 (1.82, 3.89)**	**5.36**	**3.46 (2.86, 4.20)**	–	–	1.52	**2.82 (1.97, 4.05)**	–	–	**1.67**	3.27 (2.32, 4.62)

* Per 100,000 full-time-equivalent (FTE) workers ages ≥ 15 years. Rates were calculated by NIOSH based on the number of fatalities from restricted data from the Bureau of Labor Statistics (BLS) Census of Fatal Occupational Injuries from 2011 to 2017; hours worked based on full-time-equivalent workers from the BLS Current Population Survey, 2011–2017. The views expressed here do not necessarily reflect the views of the BLS.

† Event or exposure according to the BLS Occupational Injury and Illness Classification System.

‡ Rates in bold were significantly higher than the WestON regional rate (*p*<0.05).

** Dashes indicate that data did not meet BLS publication criteria.

## Data Availability

The data that support the findings of this study are subject to third-party restrictions: Findings in this study were generated from the Bureau of Labor Statistics (BLS) Census of Fatal Occupational Injuries (CFOI) confidential research data files. A limited data set was provided to the National Institute for Occupational Safety and Health under a memorandum of understanding that strictly prohibits sharing of the data set outside of designated points of contact. Thus, the primary CFOI research file data set cannot be shared beyond NIOSH researchers approved by the Bureau of Labor Statistics. For more information on CFOI confidentiality policies, visit www.bls.gov/opub/hom/cfoi/data.htm. For more information about the CFOI data in general, visit: www.bls.gov/opub/hom/cfoi/home.htm.
